# Preparation of Whole-Cut Plant-Based Pork Meat and Its Quality Evaluation with Animal Meat

**DOI:** 10.3390/gels9060461

**Published:** 2023-06-05

**Authors:** Haodong Liu, Jinchuang Zhang, Qiongling Chen, Anna Hu, Tongqing Li, Feng Guo, Qiang Wang

**Affiliations:** 1 Institute of Food Science and Technology, Chinese Academy of Agricultural Sciences, Key Laboratory of Agro-Products Processing, Ministry of Agriculture and Rural Affairs, Beijing 100193, China; lhd1719287615@163.com (H.L.);; 2 College of Food Science and Engineering, Shanxi Agricultural University, Taigu, Jinzhong 030801, China

**Keywords:** whole-cut plant-based meat, low- and high-moisture textured vegetable proteins, mixed gel system, physicochemical properties, nutritional quality

## Abstract

Low-moisture (20~40%) and high-moisture (40~80%) textured vegetable proteins (TVPs) can be used as important components of plant-based lean meat, while plant-based fat can be characterized by the formation of gels from polysaccharides, proteins, etc. In this study, three kinds of whole-cut plant-based pork (PBP) were prepared based on the mixed gel system, which were from low-moisture TVP, high-moisture TVP, and their mixtures. The comparisons of these products with commercially available plant-based pork (C-PBP1 and C-PBP2) and animal pork meat (APM) were studied in terms of appearance, taste, and nutritional qualities. Results showed the color changes of PBPs after frying were similar to that of APM. The addition of high-moisture TVP would significantly improve hardness (3751.96~7297.21 g), springiness (0.84~0.89%), and chewiness (3162.44~6466.94 g) while also reducing the viscosity (3.89~10.56 g) of products. It was found that the use of high-moisture TVP led to a significant increase in water-holding capacity (WHC) from 150.25% to 161.01% compared with low-moisture TVP; however, oil-holding capacity (OHC) was reduced from 166.34% to 164.79%. Moreover, essential amino acids (EAAs), the essential amino acids index (EAAI), and biological value (BV) were significantly increased from 272.68 mg/g, 105.52, and 103.32 to 362.65 mg/g, 141.34, and 142.36, respectively, though in vitro protein digestibility (IVPD) reduced from 51.67% to 43.68% due to the high-moisture TVP. Thus, the high-moisture TVP could help to improve the appearance, textural properties, WHC, and nutritional qualities of PBPs compared to animal meat, which was also better than low-moisture TVP. These findings should be useful for the application of TVP and gels in plant-based pork products to improve the taste and nutritional qualities.

## 1. Introduction

The world population is expected to reach nearly 10 billion by 2050 [1], and the consumption of animal meat products will reach 470 million tons, making it difficult for the supply of animal meat to meet the demand [2]. To meet the demand for proteins, plant-based meat substitutes are beginning to attract widespread attention and are gradually becoming a hot spot for research in the food industry [3,4,5]. Plant-based meat products are protein substitutes with the texture and flavor characteristics of animal meat. Additionally, they can be regarded as a mixed gel system, which is produced by the texturization of legumes, grains, algae, and fungi [6]. This reduces the ethical issues associated with rearing and slaughtering livestock and improves animal welfare. At the same time, the production process is more environmentally friendly than conventional animal meat products [7,8], effectively avoiding greenhouse gas emissions and the waste of land and water resources associated with animal husbandry [4,9].

In recent years, more and more plant-based meat products have been accepted by consumers (omnivores, flexitarians, vegetarians, and vegans) [10]. A wide variety of products are available, such as plant-based beef burgers, bacon, ground beef, chicken nuggets, fish steaks, and ham [2]. As a complex mixed protein gel system, these products are usually produced by secondary processing using low-moisture (20~40%) textured vegetable proteins (TVPs) as an ingredient. Compared to low-moisture TVP, high-moisture (40~80%) TVP, which has a more pronounced fiber structure, can be used immediately [2]. In addition, high-moisture TVP has less nutrient loss and is sustainable [5]. However, due to high production costs and immature processes, there are few products on the market, and they are still at the experimental mapping stage [11,12]. Therefore, methods for the formation of plant-based meat products with high-moisture TVP should be investigated.

Several studies have compared plant-based meat products to animal meat from the aspects of sensory qualities, textural properties, and nutritional quality. Maria et al. [10] showed no significant distinctions in nutrient composition between plant-based meat and dairy products. Zhou et al. [13] showed that plant-based beef burgers have higher water-holding capacity and softer texture than beef burgers. Xie et al. [14,15] found that plant-based beef was less digestible than animal beef. Reynaud et al. [16] evaluated the amino acid composition of plant-based meat products using the commonly used DIAAS (digestible indispensable amino acid score) scoring model. Drewnoski et al. [17] evaluated the comprehensive nutritional quality of plant-based meat products by analyzing their nutrient composition using the NRF (nutrient rich food index). However, the plant-based meat products involved in these studies were based on low-moisture TVP, with few studies were related to high-moisture TVP.

In this study, a mixed protein gel system was prepared to simulate whole-cut plant-based pork (PBP). Whole-cut plant-based pork1 (PBP1) was prepared based on low-moisture TVP, whole-cut plant-based pork2 (PBP2) was prepared based on low-moisture TVP and high-moisture TVP mixtures, and whole-cut plant-based pork3 (PBP3) was prepared based on high-moisture TVP, all of which were compared with commercially available plant-based pork (C-PBP) and animal pork meat (APM) and collectively described in terms of appearance, nutritional composition, texture properties, water-holding capacity (WHC), oil-holding capacity (OHC), in vitro protein digestibility (IVPD), and amino acid compositions. The goal was to develop new plant-based meat products and compare their nutritional qualities with those of commercially available products to identify gaps, which should provide a theoretical basis and practical experience for the industrial production of plant-based meat products.

## 2. Results and Discussion

### 2.1. Appearance

**Figure 1:**
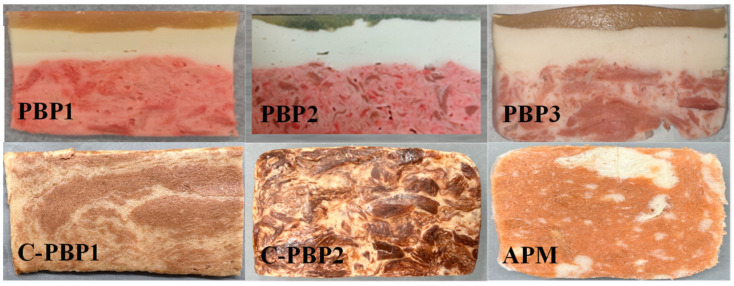
Appearance of lab products (PBP1, PBP2, and PBP3) and commercially available products (C-PBP1, C-PBP2, and APM).

The whole-cut plant-based pork meat was prepared as shown in Figure 1 and was similar to pork in terms of appearance, especially PBP3 which was made from high-moisture extruded TVP. In order to facilitate comparison of the relevant quality characteristics between animal pork and whole-cut plant-based pork, the fried method was chosen to process the samples [13]. Table 1 shows that the lightness (*L*) of five PBPs significantly decreased from 46.53~62.82 to 32.84~44.77, a* significantly increased from 14.79~24.71 to 15.09~31.17, b* changed from 15.92~20.66 to 13.21~20.80, and Δ*E* significantly increased from 40.05~48.99 to 57.46~60.14. The lightness of APM significantly decreased from 47.90 to 42.47, a* significantly increased from 19.49 to 20.95, b* changed from 16.27 to 15.99, and Δ*E* significantly increased from 48.67~53.60 to 57.46~60.14. The color coordinates of the five PBPs and their changes before and after frying were similar to APM. It was reported that the color change pattern of plant-based beef after frying was similar to that of animal beef [13,18]. These results indicate that the color of animal pork can be accurately simulated by plant-based pork products during the frying process.

### 2.2. Textural Properties

**Figure 2:**
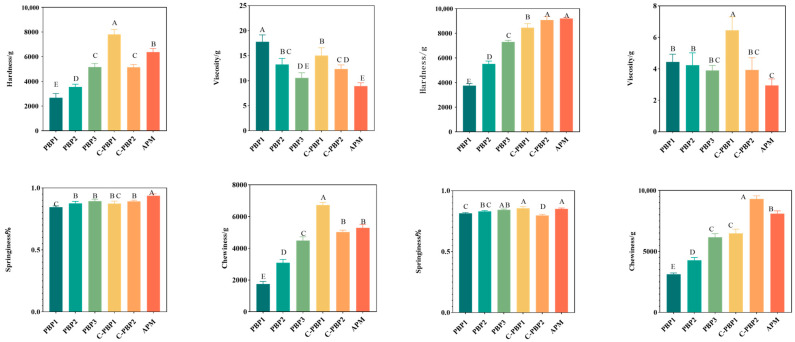
Textural properties of PBPs (PBP1, PBP2, PBP3, C-PBP1, and C-PBP2) and APM before and after frying. Different letters indicate significant differences between different products (*p* < 0.05).

Textural characteristics can be regarded as one of the most important factors influencing consumer acceptance. Therefore, this study analyzed the effect of frying treatment on the textural properties of different masses by compressing the samples twice using a textured profile analysis model. Figure 2 shows the data before and after frying. Before frying, the textural properties of the five PBPs were significantly different from each other, with hardness ranging from 2680.13 g to 7809.12 g, viscosity ranging from 12.34 g to 17.78 g, springiness ranging from 0.84% to 0.89%, and chewiness ranging from 1755.42 g to 6731.66 g. However, the textural properties of the five PBPs were significantly different from APM. After frying, there were still differences in the texture of the five PBPs, with hardness ranging from 3751.96 g to 9077.24 g, viscosity ranging from 3.89 g to 6.45 g, springiness ranging from 0.80% to 0.86%, and chewiness ranging from 3162.44 g to 9303.22 g. However, the hardness, viscosity, and springiness of PBP3, C-PBP1, and C-PBP1 were similar to APM. This may be related to protein deformation, polysaccharide breakdown, and disruption of the gel system [19,20]. Giang [13,18] et al. found that both plant-based beef burgers and animal beef burgers had similar textural changes after being fried, while plant-based beef was softer than animal beef, which was consistent with the results of this study. Furthermore, it was found that the addition of plant-based ingredients improved the firmness, consistency, and elasticity of meat analogs [21]. The hardness and viscosity of PBP3 were similar to C-PBP2, and its springiness was similar to C-PBP1. Moreover, compared to PBP1, the hardness, springiness, and chewiness of PBP3 was increased from 2680.13~3751.96 g to 5160.03~7297.21 g, 0.81~0.84% to 0.84~0.89%, and 1755.42~3162.44 g to 4492.77~6466.94 g, respectively, while its viscosity decreased from 4.44 ~17.78 g to 3.89~10.56 g. The hardness, springiness, and chewiness of PBP1, PBP2, and PBP3 were lower than that of APM. All in all, the textural properties of PBP3 were closest to those of APM, showing that high-moisture TVP was beneficial to quality improvement in PBPs. Zhang and Osen [22,23] also found that high-moisture TVP had better textural degree and springiness than low-moisture TVP.

### 2.3. WHC and OHC

**Figure 3:**
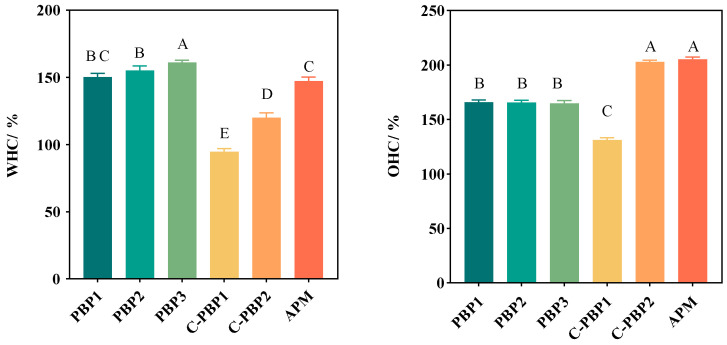
The WHC and OHC of PBPs (PBP1, PBP2, PBP3, C-PBP1, and C-PBP2) and APM. WHC stands for water-holding capacity, and OHC stands for oil-holding capacity. Different letters indicate significant differences between different products (*p* < 0.05).

It was reported that the juiciness of meat substitutes was closely related to their WHC and OHC [13]. The WHC of the five PBPs (94.75% to 161.01%) was significantly different to that of APM (147.24%), and PBP3 (made from high-moisture TVP) showed the best water-holding capacity, as shown in Figure 3. It was found that the WHC of plant-based beef burgers (94 ± 4%) was higher than that of beef burgers (88 ± 3%) [13]. This difference may be related to protein network structures, ionic strength, and hydrophilic groups. A loose protein spatial network structure and low ionic strength led to higher WHC [14]. Zhang et al. [19] studied the preparation of plant-based pork skin and found that soy protein concentrate, soy isolate, and soy oil would line up into a three-dimensional network structure to retain water. Cornet et al. [24] found that increased ionic strength resulted in lower WHC in meat substitutes. Shubham et al. [25] found that the WHC of low moisture extruded meat substitutes prepared with pea protein was associated with porous structure and hydrophilic moieties. The OHC of the five PBPs (131.34–202.97%) was significantly different from that of APM (205.06%). It was more influenced by surface hydrophobic groups and spatial structure, with the more exposed hydrophobic groups improving oil retention properties [15,19]. The addition of high-moisture TVP significantly improved WHC from 150.25% (PBP1) to 161.01% (PBP3) but decreased OHC from 166.34% (PBP1) to 164.79% (PBP3), which should be further improved to the level found in C-PBP2 (202.97%).

### 2.4. Nutritional Composition

Table 2 shows the nutritional compositions of the PBPs and APM. The fat content of the five PBPs ranged from 4.14 to 6.54 g/100 g, which was lower than that of APM (11.91 g/100 g). It has been reported that plant-based meat is lower in fat, cholesterol-free, and can significantly reduce the incidence of cardiovascular disease [6,26]. The ash content (1.85~1.96 g/100 g) of PBP3, C-PBP1, and C-PBP2 was similar to APM (1.91 g/100 g), indicating that the content of mineral elements in the samples was comparable to that of inorganic compounds after high-temperature oxidation. Protein content ranged from 6.04 to 18.22 g/100 g, and the protein content of PBP2, PBP3, and C-PBP2 was more than 12 g/100 g, which means they can thus be categorized as high-protein foods [6,27,28]. Moreover, the protein content of PBP3 and C-PBP2 was similar to that of APM (16.79 g/100 g). The moisture content of PBPs was between 56.12 and 63.31 g/100 g, which was closer to APM (58.83 g/100 g). Dietary fiber, as a characteristic ingredient of PBPs, ranged from 1.96 to 5.02 g/100 g, which could increase satiety, combine low density lipoprotein (LDL) in organic compounds, accelerate probiotic reproduction, and improve the body’s antioxidant capacity [29,30]. Moreover, the PBPs contained more iron content (1.22~3.90 mg/100 g) than APM (0.85 mg/100 g), and the PBPs also contained more calcium content (15.24~48.57 mg/100 g) than APM (5.60 mg/100 g). The addition of high-moisture TVP was found to enhance the basic nutrient composition. Moreover, these ingredients not only provide rich nutrients, but also play an important role in improving textural properties, including the hardness, springiness, and chewiness of the products [27]. Wang et al. [31] found that plant-based meat with lower protein and higher moisture content was softer with less chewiness [32], and plant-based meat with higher fat content had better springiness.

### 2.5. IVPD

**Figure 4:**
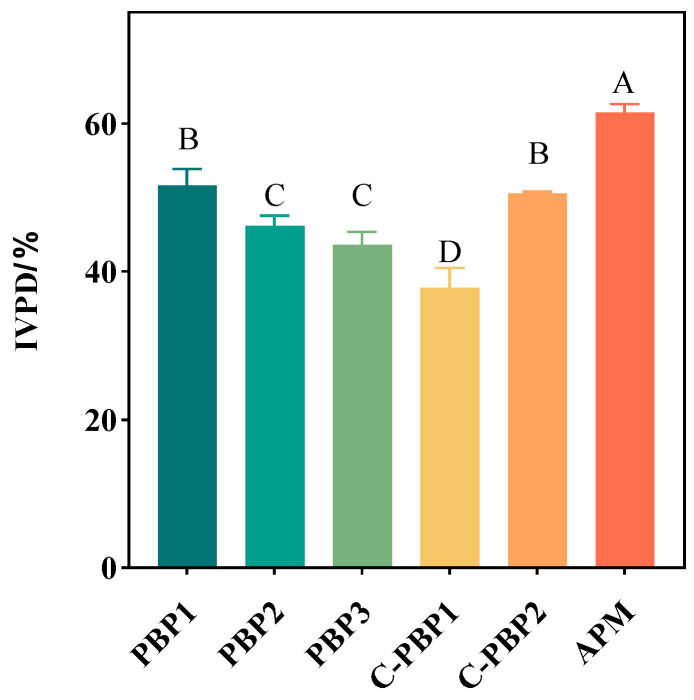
In vitro protein digestibility of PBPs (PBP1, PBP2, PBP3, C-PBP1, and C-PBP2) and APM. IVPD stands for in vitro protein digestibility. Different letters indicate significant differences between different products (*p* < 0.05).

Figure 4 shows that the IVPD of the five PBPs (ranging from 37.87% to 51.67%) was significantly lower than that of APM (61.49%), which may be due to the different types and structures of proteins, including α-helix and β-fold structures, total sulfhydryl content, and surface hydrophobicity. Most of the plant proteins were globulins or hemispheric proteins, and the activated sites in contact with proteases were wrapped inside, which made it difficult for them to be broken down by enzymes [15,33]. On the contrary, animal proteins were mostly irregular chain strips, and more activated sites were exposed to make protease work better, which could degrade the protein into peptide chains, short peptides, and amino acids [15,16]. It was found that beef showed higher digestibility than plant-based beef [14], which was also illustrated by the relationship between digestibility and the secondary structure of the protein. With the addition of high-moisture TVP, its digestibility gradually decreased, which may be related to its increasing protein content because the protein content of high-moisture TVP was higher than that of low-moisture TVP [9,12]. Therefore, the globular proteins of PBP3 were not easily broken down by protease. In addition, increasing carbohydrate content such as dietary fibers can decrease protein digestibility. Ahmad et al. [14,15,34] found that dietary fibers can interact with proteins to form complexes through hydrogen bonding and van der Waals forces, which will inhibit protein hydrolysis. An earlier study [35] reported that dietary fibers can also influence the effect of digestive enzymes by altering their activity, which in turn slows down the rate of protein digestion. Zhou et al. [14] found that plant-based meat weakens pepsin activity, leading to reduced digestibility. These studies could better explain the results of this experiment. The results also suggested that high-moisture TVP can alter the digestive characteristics of plant-based meat, and Cho et al. [21] also found that the digestibility of high-moisture TVP can be decreased.

### 2.6. Nutritional Evaluation of Amino Acids

PBPs can be viewed as new alternative protein foods, and amino acid patterns are an important factor in assessing their nutritional quality. In this study, essential amino acids (EAAs), non-essential amino acids (NEAAs), total amino acids (TAAs), the EAAI, and BV were analyzed. The TAA content (809.44–1126.46 mg/g) of PBP1, PBP2, PBP3, and C-PBP2 was not significantly different from that of APM (1012.70 mg/g), but the EAA content (122.88~402.66 mg/g), NEAA content (342.16~723.79 mg/g), and EAA/NEAA ratio (35.91~55.63%) of PBPs were significantly different to APM. Moreover, the EAA and NEAA content of PBP1, PBP2, and C-PBP1 was lower than that of APM. Chen et al. [36] found that chicken substitutes contained less essential and non-essential amino acids than chicken, and the conclusion reached was consistent with the results of the present study. Several studies [31,33,37] have shown that the amino acid composition of high-quality proteins is about 60% and 40% for EAA/NEAA and EAA/TAA ratio, and the above results suggest that there is still a gap between the amino acid pattern of PBPs and APM.

The EAAI was also often used to evaluate the quality of food proteins, where an EAAI value > 95 indicates a superior protein source, 86 < EAAI ≤ 95 indicates a good protein source, 76 < EAAI ≤ 86 indicates a usable protein source, and EAAI ≤ 75 indicates an unsuitable protein source. Table 3 shows that PBP1, PBP2, PBP3, and C-PBP2 were superior protein sources, and the EAAI of C-PBP2 (153.09) was even better than that of APM (149.64).

The BV can indicate the degree of utilization for the protein in food after digestion and absorption. Table 3 shows that the BV of PBP1, PBP2, PBP3, and C-PBP2 was greater than 100, indicating that these four PBPs showed a high level of bioavailability. The highest BV of C-PBP2 was 155.17, which was higher than APM (151.40). Moreover, with the addition of high-moisture TVP, TAAs, EAAs, EAA/NEAA, EAA/TAA, the EAAI, and the BV of PBPs increased continuously, indicating that the addition of high-moisture TVP was capable of changing the amino acid pattern and reducing the gap to animal meat, thus showing that high-moisture TVP had more nutritional advantages than low-moisture TVP. It has been reported that high-moisture TVP can release more free amino acids and have a more balanced amino acid pattern, which is consistent with the results of this study [33,38].

The AASs (Table 4) and CSs (Table 5) were also compared. It was shown that lysine and leucine were the first- and second-limiting amino acids of PBP1 and PBP2, while PBP3 and C-PBP2 did not contain any limiting amino acids, with AASs between 116.01~297.63 and 127.12~345.30. According to the standard of the egg protein patterns, PBP1, PBP2, and C-PBP1 were significantly different from APM (101.80–168.00), with threonine (28.00–85.49) and lysine (31.48–94.92) especially lower than in APM (118.75 and 168.00). With the addition of high-moisture TVP, AASs increased from 80.08~252.51 to 116.01~297.63 and CSs increased from 64.56~159.93 to 85.49~188.50, indicating that high-moisture TVP was beneficial to improvement in the AASs and CSs of plant-based meat, owing to the superior amino acid composition ratio and higher amino acid and chemical scores of PBP3 compared to PBP1.

## 3. Conclusions

In this study, three kinds of whole-cut PBPs were prepared based on the mixed gel system, which were from low-moisture TVP, high-moisture TVP, and their mixtures, respectively. These PBPs were compared with two commercially available plant-based pork products and APM in terms of appearance, taste, and nutritional qualities. The results show that the color changes in PBPs were significant after frying, with changes consistent with those in APM. The addition of high-moisture TVP significantly improved the hardness (3751.96~7297.21 g), springiness (0.84~0.89%), and chewiness (3162.44~6466.94 g) of products while also reducing viscosity (3.89~10.56 g). It was also found that WHC was significantly increased from 150.25% to 161.01% due to the high-moisture TVP; however, OHC was slightly reduced from 166.34% to 164.79%. Moreover, the EAA content, EAAI, and BV of PBPs were significantly increased to 362.65 mg/g, 141.34, and 142.36, respectively, because of the high-moisture TVP; however, in vitro protein digestibility (IVPD) was reduced from 51.67% to 43.68%. Therefore, high-moisture TVP can be added to improve the properties of PBPs better than low-moisture TVP. These findings should be useful for the application of TVP and gels in plant-based pork products to improve nutritional qualities. In the future, this will contribute to the establishment of quality evaluation standards for plant-based meat products and help consumers understand the quality characteristics of plant-based meat products.

## 4. Materials and Methods

### 4.1. Materials

Soy protein isolate (SPI) was supplied by Yihai Kerry Co., Ltd. (Shanghai, China), containing 90.81% protein (dry basis), 5.55% moisture, 0.36% fat (dry basis), and 4.67% ash content (dry basis). Wheat gluten (WG) was supplied by Yihai Kerry Co., Ltd. (Shanghai, China), containing 87.59% protein (dry basis), 8.28% moisture, 1.59% fat (dry basis), and 0.81% ash content (dry basis). Peanut protein powder (PPP) was supplied by Changshou Food Co., Ltd., (Qingdao, China), containing 54.88% protein (dry basis), 5.91% moisture, 5.31% fat (dry basis), and 4.39% ash content (dry basis). Sunflower oil, sweet potato starch, transglutaminase (TG), and soy sauce were purchased from Beijing Cui Feng Technology Co., Ltd. (Beijing, China). The curdlan gum was provided by Zhengzhou Opel Biotechnology Co., Ltd. (Zhengzhou, China). Pork flavoring powder Y20076V was provided by Beijing Hongxi Professional Technology Co., Ltd. (Beijing, China). Low-moisture TVP was provided by Zhejiang Baichuan Food Co., Ltd. (Wenzhou, China). The high-moisture TVP was prepared based on our previous experiments [22]. Trypsin (1:250U/mg) and pepsin (1:250U/mg) were purchased from Beijing Solarbio Technology Co., Ltd. (Beijing, China). Sulian plant-based pork was purchased from Ningbo Sulian Food Co., Ltd. (Ningbo, China). The new vegetarian pork was purchased from Hangzhou Plant Meat Health Technology Co., Ltd. (Hangzhou, China) and the two commercial plant-based pork products were named C-PBP1 and C-PBP2. The animal pork meat (APM) was purchased from Changsha Xiaohu Food Co., Ltd. (Beijing, China).

### 4.2. Preparation of Plant-Based Pork

The plant-based pork products were prepared by the following steps:

(1) The low-moisture TVP was soaked in the pigment solution (0.50% soy sauce solution) for 20 min, then the soaked low-moisture TVP and high-moisture TVP were separated into filaments.

(2) The wheat gluten (1.00%), curdlan gum (2.50%), and water were combined to form a mixture with uniform texture to simulate the plant-based pork skin gels. The wheat gluten (5.00%), curdlan gum (5.00%), sweet potato starch (10.00%), sunflower oil (10.00%), TG enzyme (1.00%), pork flavoring powder (0.20%), and water were mixed well in a mixer to form a mixture with uniform texture to simulate the plant-based fat gels (named A). The mixer model was a QSJ-B03L5, which was purchased from Foshan Xiaoxiong Technology Co., Ltd. The speed of the mixer was 1000 rpm/min, and the mixing time was three minutes.

(3) In this study, the protein gels formed by mixing the plant-based pork mixture, TVP, and A formed a mixed gel system to simulate the plant-based pork. A with weight M and low-moisture TVP with weight M were mixed and heated in a water bath (80 °C for 145 min) and cooled (0~4 °C) for 6 h to form PBP1. Weight M of A and weight M/2 of low-moisture TVP and weight M/2 of high-moisture TVP were mixed and heated in a water bath (80 °C for 145 min) and cooled (0~4 °C) for 6 h to form PBP2. Weight M of A and weight M of high-moisture TVP were mixed and heated in a water bath (80 °C for 145 min) and cooled (0~4 °C) for 6 h to form PBP3. The three PBPs were from different mixed gels.

(4) PBP1, PBP2, PBP3, C-PBP1, C-PBP2, and APM were fried in a pan for 120 s and then turned and fried for 60 s, with the central temperature maintained above 70 °C.

### 4.3. Nutritional Composition

Moisture content was analyzed using AOAC method 930.15. Fat was determined using AOAC method 920.39 [39]. Fiber content was determined using the method of [40]. Protein content was determined according to the Kjeldahl method (FOSS KJELTEC 2300, Copenhagen, Denmark) and ash content was analyzed using the method of [25]. The amino acid profiles were analyzed following AOAC method 994.12 [39]. Determination of elemental sodium, calcium, and iron content referred to the method of [41]. At least five measurements were made for each sample data point, with the mean value and standard deviation then calculated and significance analysis conducted. It is important to note that some of the data are estimates, which while not reflecting the true values do reflect the nutritional properties of the food.

### 4.4. Appearance

The color values of the samples of mixed gels were determined by a colorimeter (CS-600, CHN Spec, Hangzhou, China). The samples were placed on the surface of a white standard plate, and lightness (*L*), redness (*a*), and yellowness (*b*) were measured, with measurements repeated at least five times. The standard *L*_0_, *a*_0_, and *b*_0_ values of the calibration plate were 89.73, −0.78, and 1.88. The total color difference (Δ*E*) of the samples was calculated by Equation (1) below. At least five measurements were made for each sample data point, with the mean value and standard deviation then calculated and significance analysis conducted.

(1) $$\Delta E=\sqrt{{\left(L-L_{0}\right)}^{2}+{\left(a-a_{0}\right)}^{2}+{\left(b-b_{0}\right)}^{2}}$$

### 4.5. Texture Profile Analysis

The texture of the mixed gels was measured with a TA.XT2 Texture Analyzer (Stable Micro Systems, London, UK) according to our previous methods [20]. Hardness, viscosity, springiness, and chewiness were recorded using a P/36R probe (cylinder, ∅36 mm). The operating parameters were 2.00 mm/s pre-velocity, 1.00 mm/s mid-velocity, 2.00 mm/s post-velocity, and 50% compression deformation. All of the determinations were repeated 5 times and averaged. At least ten measurements were made for each sample data point, with the mean value and standard deviation then calculated and significance analysis conducted.

### 4.6. WHC and OHC

The method used to measure the WHC of samples was based on a simple low-speed centrifugation method described previously [13]. In a centrifuge tube, 1.0 g of sample was mixed with 10 mL of distilled water. The samples were centrifuged for 25 min at a centrifugal force of 1000× *g* while the temperature was set at 20 °C. The supernatants were then removed. The *WHC* of the sample was calculated by Equation (2) below, where *M*_0_ indicates the sample weight, *M*_1_ the total mass of the sample and centrifuge tube before water absorption (g), and *M*_2_ the total mass of the sample and centrifuge tube after water absorption (g). At least five measurements were made for each sample data point, with the mean value and standard deviation then calculated and significance analysis conducted.

(2) $$WHC\%=\frac{M_{2}-M_{1}}{M_{0}}\times 100\%$$

In a centrifuge tube, 1.0 g of the sample was mixed with 10 mL of sunflower oil. The samples were centrifuged for 30 min at a centrifugal force of 2000× *g* while the temperature was set at 20 °C. The supernatants were then removed. The *OHC* of the sample was calculated by Equation (3) below, where *N*_0_ indicates the sample weight, *N*_1_ the total mass of the sample and centrifuge tube before water absorption (g), and *N*_2_ the total mass of the sample and centrifuge tube after water absorption (g). At least five measurements were made for each sample data point, with the mean value and standard deviation then calculated and significance analysis conducted.

(3) $$OHC\%=\frac{N_{2}-N_{1}}{N_{0}}\times 100\%$$

### 4.7. IVPD

A volume of 6.1 mL of concentrated hydrochloric acid was dissolved into 1000 mL of distilled water (PH 2.0), with 0.8 g of pepsin (250 U/mg) then dissolved to obtain a pepsin solution with an enzyme activity of 200 U/mL. Subsequently, 3.521 g of sodium dihydrogen phosphate (NaH_2_PO_4_·2H_2_O) was dissolved into 100 mL of distilled water to obtain a sodium dihydrogen phosphate solution with a concentration of 0.2 mol/L. Following this, 71.64 g of disodium hydrogen phosphate (Na_2_HPO_4_-12H_2_O) was dissolved into 1000 mL of distilled water to obtain a disodium hydrogen phosphate solution with a concentration of 0.2 mol/L. A volume of 53 mL of 0.2mol/L sodium dihydrogen phosphate solution and 947 mL of 0.2 mol/L disodium hydrogen phosphate solution were then mixed to prepare 1000 mL of a 0.2 mol/L phosphate buffer solution (pH 8.0), with 0.6g of trypsin (250 U/mg) then dissolved to obtain a trypsin solution with an enzyme activity of 150 U/mL.

To perform the experiment, 1.0 g of finely crushed sample was taken to the tube and carefully weighed to an accuracy of 0.0001 g. Subsequently, 10 mL of pepsin solution (200 U/mL) at pH 2.0 was added, and it was placed in a constant temperature incubator at (37 ± 1) °C and shaken at 190 r/min. After 3 h, the tube was removed, and 2.0 mL of 0.5 mol/L NaOH solution and 30 mL of pepsin solution (150 U/mL) at pH 8.0 were added. The centrifuge tube was then removed, and 10 mL of 10% TCA solution was added. The tube was shaken well and left for 1 h. It was then centrifuged at 4 °C (centrifugal force 4800× *g*) for 10 min. The supernatant was collected, and protein content was determined using the Kjeldahl method. To ensure accuracy, a blank control group was also prepared under the same conditions. The *IVPD* of the samples was calculated by Equation (4) below. *A*_0_ represents protein content in the sample, *A*_1_ represents protein content in the upper liquid, and *A*_2_ represents protein content in the blank control group. At least five measurements were made for each sample data point, with the mean value and standard deviation then calculated and significance analysis conducted.

(4) $$IVPD\%=\frac{A_{1}-A_{2}}{A_{0}}\times 100\%$$

### 4.8. Nutritional Evaluation of Amino Acids

The amino acid score (*AAS*), chemical score (*CS*), essential amino acid index (*EAAI*), and biological value (*BV*) were calculated according to scoring methods recommended by the FAO/WHO [42]. The formulae are as follows.

(5) $$AAS=\frac{A_{00}}{A_{S}}$$

(6) $$CS=\frac{A_{00}}{A_{S1}}$$

(7) $$EAAI=100\times \sqrt[{n}]{\frac{T\mathrm{hr}}{T{\mathrm{hr}}_{s}}\times \frac{V\mathrm{al}}{V{\mathrm{al}}_{s}}\times \frac{(\mathrm{Met}+\mathrm{Cys})}{{(\mathrm{Met}+\mathrm{Cys})}_{s}}\cdots \times \frac{L\mathrm{ys}}{L{\mathrm{ys}}_{s}}}$$

(8) BV=1.09×EAAI−11.7

*A*_00_ represents the amino acid content of the sample, *A_S_* represents the FAO/WHO model reference value, *A_S_*_1_ represents the egg protein model reference value, *s* represents the FAO/WHO model reference value, and n represents the number of essential amino acids to be measured.

### 4.9. Statistical Analysis

All data were analyzed using analysis of variance (ANOVA) in the general linear model procedure of the Statistical Product and Service Solutions software package (SPSS, version 19.0, IBM, Chicago, IL, USA). The differences between group means were analyzed using Duncan’s multiple range test. Statistical significance was set at a 0.05 probability level.

## Figures and Tables

**Table 1:** Effect of frying on the appearance of PBPs (PBP1, PBP2, PBP3, C-PBP1, and C-PBP2) and APM.

Cooker	Indicator	PBP1	PBP2	PBP3	C-PBP1	C-PBP2	APM
Before frying	*L*	62.82 ± 0.54 ^A^	53.03 ± 0.46 ^C^	54.99 ± 0.59 ^B^	46.53 ± 0.53 ^D^	47.93 ± 0.82 ^D^	47.90 ± 0.63 ^D^
	a*	24.71 ± 0.57 ^A^	19.33 ± 0.37 ^B^	16.85 ± 0.46 ^C^	14.79 ± 0.43 ^D^	15.84 ± 0.51 ^CD^	19.49 ± 0.51 ^B^
	b*	17.05 ± 0.40 ^C^	15.92 ± 0.04 ^C^	20.66 ± 0.47 ^A^	18.91 ± 0.58 ^B^	19.08 ± 0.15 ^B^	16.27 ± 0.92 ^C^
	∆*E*	40.05 ± 0.05 ^C^	44.14 ± 0.28 ^B^	43.25 ± 0.46 ^B^	48.99 ± 0.55 ^A^	48.16 ± 0.86 ^A^	48.67 ± 0.49 ^A^
After frying	*L*	44.77 ± 1.49 ^A^	39.46 ± 1.15 ^C^	36.74 ± 0.51 ^D^	32.84 ± 0.31 ^E^	38.51 ± 0.30 ^CD^	42.47 ± 0.46 ^B^
	a*	31.17 ± 0.11 ^A^	24.40 ± 1.17 ^B^	17.85 ± 1.63 ^D^	15.09 ± 0.31 ^E^	18.50 ± 0.36 ^D^	20.95 ± 0.32 ^C^
	b*	20.80 ± 0.21 ^A^	19.13 ± 0.54 ^AB^	17.23 ± 1.53 ^BC^	13.21 ± 0.20 ^D^	19.36 ± 0.25 ^A^	15.99 ± 0.21 ^C^
	∆*E*	58.32 ± 1.12 ^B^	58.82 ± 0.58 ^AB^	58.27 ± 0.63 ^B^	60.14 ± 0.25 ^A^	57.46 ± 0.22 ^B^	53.60 ± 0.58 ^C^

Note: Letters indicate significant differences between different products (*p* < 0.05). PBP1 indicates whole-cut plant-based pork1, PBP2 indicates whole-cut plant-based pork2, PBP3 indicates whole-cut plant-based pork3, C-PBP1 and C-PBP2 indicate two commercially available plant-based pork products, and APM indicates animal pork meat.

**Table 2:** The nutrient composition of PBPs (PBP1, PBP2, PBP3, C-PBP1, and C-PBP2) and APM.

Content	PBP1	PBP2	PBP3	C-PBP1	C-PBP2	APM
Fat g/100 g	4.14 ± 0.16 ^D^	4.23 ± 0.08 ^CD^	4.98 ± 0.31 ^C^	6.54 ± 0.24 ^B^	4.52 ± 0.01 ^CD^	11.91 ± 0.19 ^A^
Ash g/100 g	0.84 ± 0.02 ^B^	0.96 ± 0.16 ^B^	1.85 ± 0.03 ^A^	1.96 ± 0.27 ^A^	1.96 ± 0.05 ^A^	1.91 ± 0.00 ^A^
Protein g/100 g	10.69 ± 0.18 ^D^	13.36 ± 0.15 ^C^	16.43 ± 0.25 ^B^	6.04 ± 0.28 ^E^	18.22 ± 0.93 ^A^	16.79 ± 0.22 ^B^
Moisture g/100 g	63.31 ± 0.36 ^A^	58.57 ± 1.00 ^B^	56.12 ± 0.75 ^C^	58.59 ± 0.89 ^B^	57.80 ± 0.78 ^B^	58.83 ± 0.62 ^A^
Dietary fiber g/100 g	4.02 ± 0.12 ^C^	4.62 ± 0.31 ^B^	5.02 ± 0.44 ^A^	3.81 ± 0.25 ^C^	1.96 ± 0.35 ^D^	—
Iron mg/100 g	1.22 ± 0.11 ^D^	1.74 ± 0.08 ^C^	1.99 ± 0.20 ^B^	1.22 ± 0.14 ^D^	3.90 ± 0.35 ^A^	0.85 ± 0.09 ^E^
Sodium mg/100 g	266.94 ± 65.85 ^D^	510.96 ± 33.45 ^C^	878.54 ± 78.51 ^A^	838.01 ± 45.95 ^A^	551.25 ± 66.57 ^C^	780.00 ± 7.30 ^B^
Calcium mg/100 g	15.51 ± 2.00 ^D^	27.11 ± 1.84 ^C^	30.53 ± 2.54 ^B^	15.24 ± 1.99 ^D^	48.57 ± 3.58 ^A^	5.60 ± 1.25 ^E^

Note: Letters indicate significant differences between different products (*p* < 0.05); “-” indicates not tested. Cholesterol content reference for the study [32]. PBP1 indicates whole-cut plant-based pork1, PBP2 indicates whole-cut plant-based pork2, PBP3 indicates whole-cut plant-based pork3, C-PBP1 and C-PBP2 indicate two commercially available plant-based pork products, and APM indicates animal pork meat.

**Table 3:** Amino acid composition and nutritional evaluation of PBPs (PBP1, PBP2, PBP3, C-PBP1, and C-PBP2) and APM.

Amino Acids (mg/g Protein)	PBP1	PBP2	PBP3	C-PBP1	C-PBP2	APM
THR *	25.83 ± 0.85 ^E^	30.64 ± 0.21 ^D^	34.20 ± 0.31 ^C^	11.22 ± 0.55 ^F^	44.98 ± 0.36 ^B^	47.50 ± 0.71 ^A^
VAL *	43.17 ± 0.29 ^D^	48.02 ± 0.57 ^C^	56.13 ± 0.68 ^A^	19.72 ± 0.17 ^E^	57.00 ± 0.25 ^A^	50.90 ± 0.44 ^B^
MET *	13.69 ± 0.15 ^E^	15.35 ± 0.22 ^D^	17.556 ± 0.39 ^C^	7.91 ± 0.26 ^F^	21.58 ± 0.51 ^A^	19.9 ± 0.61 ^B^
ILE *	36.03 ± 1.57 ^D^	41.57 ± 0.39 ^C^	46.83 ± 0.57 ^B^	18.46 ± 0.61 ^E^	52.00 ± 0.39 ^A^	47.70 ± 0.78 ^B^
LEU *	57.87 ± 0.53 ^E^	68.97 ± 1.05 ^D^	79.45 ± 1.26 ^B^	20.49 ± 0.18 ^F^	75.00 ± 0.27 ^C^	82.10 ± 0.48 ^A^
PHE *	50.57 ± 0.28 ^D^	54.31 ± 0.41 ^C^	62.3 ± 0.73 ^B^	23.78 ± 0.29 ^F^	65.00 ± 0.56 ^A^	39.80 ± 0.67 ^E^
LYS *	36.04 ± 0.94 ^E^	44.04 ± 0.23 ^D^	52.21 ± 0.34 ^C^	17.32 ± 1.05 ^F^	77.10 ± 0.26 ^B^	92.40 ± 0.38 ^A^
TRP *	9.50 ± 0.31 ^B^	10.00 ± 0.62 ^B^	14.00 ± 0.74 ^A^	4.00 ± 0.35 ^C^	10.00 ± 0.69 ^B^	13.00 ± 0.84 ^A^
ASP	86.50 ± 0.16 ^C^	94.39 ± 0.24 ^B^	103.92 ± 0.43 ^A^	32.10 ± 0.18 ^E^	82.50 ± 0.27 ^D^	93.80 ± 0.48 ^B^
SER	43.06 ± 1.11 ^D^	48.40 ± 0.27 ^C^	55.42 ± 0.40 ^B^	17.72 ± 0.72 ^F^	72.90 ± 0.47 ^A^	28.80 ± 0.92 ^E^
GLU	119.90 ± 0.37 ^F^	131.50 ± 0.74 ^E^	152.60 ± 0.88 ^A^	135.55 ± 0.22 ^D^	150.10 ± 0.32 ^B^	148.30 ± 0.57 ^C^
GLY	37.38 ± 0.20 ^E^	45.19 ± 0.29 ^D^	47.43 ± 0.51 ^C^	14.12 ± 0.34 ^F^	57.30 ± 0.66 ^B^	63.90 ± 0.80 ^A^
ALA	32.44 ± 2.04 ^E^	35.65 ± 0.50 ^D^	39.90 ± 0.74 ^C^	14.39 ± 0.78 ^F^	50.62 ± 0.51 ^B^	61.80 ± 1.02 ^A^
TYR	45.39 ± 0.69 ^C^	44.85 ± 1.36 ^C^	50.80 ± 1.63 ^B^	21.86 ± 0.24 ^E^	66.22 ± 0.35 ^A^	36.70 ± 0.63 ^D^
HIS	18.77 ± 0.36 ^E^	22.00 ± 0.54 ^D^	24.15 ± 0.95 ^C^	9.19 ± 0.37 ^F^	33.31 ± 0.73 ^B^	43.90 ± 0.87 ^A^
ARG	81.64 ± 1.22 ^D^	93.00 ± 0.30 ^C^	105.00 ± 0.44 ^B^	58.00 ± 1.36 ^F^	110.87 ± 0.33 ^A^	71.40 ± 0.50 ^E^
PRO	52.70 ± 0.41 ^D^	60.95 ± 0.81 ^C^	72.45 ± 0.97 ^B^	31.25 ± 0.46 ^E^	75.00 ± 0.90 ^A^	51.60 ± 1.08 ^D^
CYS	19.00 ± 0.21 ^B^	20.00 ± 0.319176 ^B^	20.00 ± 0.56 ^B^	8.00 ± 0.24 ^C^	25.00 ± 0.35 ^A^	19.20 ± 0.63 ^B^
TAA	809.44 ± 72.11 ^AB^	908.80 ± 90.15 ^AB^	1034.32 ± 152.30 ^AB^	465.05 ± 52.36 ^C^	1126.46 ± 136.50 ^A^	1012.70 ± 147.90 ^AB^
EAA	272.68 ± 15.6 ^D^	312.875 ± 9.6 ^C^	362.65 ± 11.5 ^B^	122.88 ± 2.9 ^E^	402.66 ± 5.63 ^A^	393.3 ± 7.51 ^A^
NEAA	536.755 ± 35.11 ^D^	595.925 ± 15.6 ^C^	671.66 ± 26.36 ^AB^	342.16 ± 5.6 ^E^	723.79 ± 6.55 ^A^	619.4 ± 12.11 ^BC^
EAA/NEAA	50.80 ± 0.01% ^E^	52.50 ± 0.01% ^D^	53.99 ± 0.13% ^C^	35.91 ± 0.02% ^F^	55.63 ± 0.14% ^B^	63.50 ± 0.02% ^A^
EAA/TAA	33.69 ± 0.01% ^D^	34.43 ± 0.14% ^C^	35.06 ± 0.02% ^B^	26.42 ± 0.01% ^E^	35.75 ± 0.01% ^B^	39.84 ± 0.01% ^A^
EAAI	105.52 ± 1.05 ^E^	120.07 ± 1.25 ^D^	141.34 ±0.39 ^C^	48.46 ± 0.77 ^F^	153.09 ± 0.51 ^A^	149.64 ± 0.88 ^B^
BV	103.32 ±0.58 ^E^	119.17 ± 0.60 ^D^	142.36 ± 1.05 ^C^	41.12 ± 0.96 ^F^	155.17 ± 1.18 ^A^	151.40 ± 1.42 ^B^

Note: “*” indicates essential amino acids. Letters indicate significant differences between different products (*p* < 0.05). PBP1 indicates whole-cut plant-based pork1, PBP2 indicates whole-cut plant-based pork2, PBP3 indicates whole-cut plant-based pork3, C-PBP1 and C-PBP2 indicate two commercially available plant-based pork products, and APM indicates animal pork meat.

**Table 4:** The amino acid scores (AASs) of PBPs (PBP1, PBP2, PBP3, C-PBP1, and C-PBP2) and APM.

Amino Acid Scores	WHO/FAO 2007	PBP1	PBP2	PBP3	C-PBP1	C-PBP2	APM
His	15.00	125.10 ± 2.42 ^E^	146.67 ± 3.57 ^D^	161.00 ± 6.34 ^C^	61.27 ± 2.47 ^F^	222.03 ± 4.87 ^B^	292.67 ± 5.83 ^A^
Thr	23.00	112.28 ± 3.69 ^E^	133.22 ± 1.40 ^D^	148.67 ± 2.06 ^C^	48.76 ± 3.66 ^F^	195.57 ± 2.40 ^B^	206.52 ± 4.73 ^A^
Lys	45.00	**80.08 ± 0.64 ^**D**^**	**97.87 ± 3.78 ^**C**^**	116.01 ± 4.53 ^A^	**38.48 ± 1.12 ^**E**^**	171.33 ± 1.65 ^B^	205.33 ± 2.93 ^A^
Leu	59.00	**98.08 ± 0.25 ^**E**^**	116.89 ± 1.48 ^D^	134.65 ± 2.64 ^C^	**34.72 ± 1.72 ^**F**^**	127.12 ± 3.40 ^B^	139.15 ± 4.08 ^A^
Ile	30.00	120.08 ± 5.23 ^D^	138.55 ± 2.58 ^C^	156.10 ± 3.81 ^B^	61.53 ± 4.03 ^E^	173.33 ± 2.64 ^A^	159.00 ± 5.20 ^B^
Met + Cys	22.00	148.59 ± 2.41 ^D^	160.66 ± 6.99 ^C^	170.70 ± 8.37 ^B^	72.32 ± 1.23 ^E^	211.73 ± 1.81 ^A^	177.73 ± 3.22 ^B^
Phe + Tyr	38.00	252.51 ± 0.73 ^D^	260.92 ± 2.74 ^C^	297.63 ± 4.87 ^B^	120.08 ± 1.90 ^F^	345.30 ± 3.74 ^A^	201.32 ± 4.48 ^E^
Val	39.00	110.69 ± 2.39 ^E^	123.12 ± 1.54 ^D^	143.91 ± 2.27 ^C^	50.56 ± 6.98 ^F^	146.15 ± 1.72 ^A^	130.51 ± 2.54 ^B^
Trp	6.00	158.33 ± 5.28 ^D^	166.67 ± 10.40 ^C^	233.33 ± 12.40 ^A^	66.67 ± 5.91 ^E^	166.67 ± 11.60 ^C^	216.67 ± 13.90 ^B^

Note: Bold indicates first- or second-limiting amino acids, and letters indicate significant differences in the same row of data. PBP1 indicates whole-cut plant-based pork1, PBP2 indicates whole-cut plant-based pork2, PBP3 indicates whole-cut plant-based pork3, C-PBP1 and C-PBP2 indicate two commercially available plant-based pork products, and APM indicates animal pork meat.

**Table 5:** The chemical scores (CSs) of PBPs (PBP1, PBP2, PBP3, C-PBP1, and C-PBP2) and APM.

Chemical Scores	FAO Pattern 1984	PBP1	PBP2	PBP3	C-PBP1	C-PBP2	APM
Thr	40.00	64.56 ± 2.12 ^E^	76.60 ± 0.52 ^D^	85.49 ± 0.77 ^C^	28.04 ± 1.37 ^F^	112.45 ± 0.90 ^B^	118.75 ± 1.77 ^A^
Lys	55.00	65.52 ± 0.52 ^E^	80.07 ± 1.03 ^D^	94.92 ± 1.23 ^C^	31.48 ± 0.30 ^F^	140.18 ± 0.45 ^B^	168.00 ± 0.80 ^A^
Leu	70.00	82.67 ± 0.21 ^E^	98.52 ± 0.31 ^D^	113.49 ± 0.56 ^B^	29.26 ± 0.37 ^F^	107.14 ± 0.73 ^C^	117.29 ± 0.87 ^A^
Ile	40.00	90.06 ± 3.92 ^D^	103.91 ± 0.97 ^C^	117.08 ± 1.43 ^B^		130.00 ± 0.99 ^A^	119.25 ± 1.95 ^B^
Met + Cys	35.00	93.40 ± 1.52 ^E^	100.99 ± 2.99 ^D^	107.30 ± 3.59 ^C^	45.46 ± 0.52 ^F^	133.09 ± 0.77 ^A^	111.71 ± 1.38 ^B^
Phe + Tyr	60.00	159.93 ± 0.46 ^D^	165.25 ± 0.68 ^C^	188.00 ± 1.21 ^B^	76.05 ± 0.47 ^F^	218.69 ± 0.93 ^A^	127.50 ± 1.12 ^E^
Val	50.00	86.34 ± 1.87 ^D^	96.03 ± 0.46 ^C^	112.25 ± 0.68 ^A^	39.44 ± 2.09 ^E^	114.00 ± 0.51 ^A^	101.80 ± 0.76 ^B^
Trp	10.00	95.00 ± 3.16 ^B^	100.00 ± 6.24 ^B^	140.00 ± 7.48 ^A^	40.00 ± 3.54 ^C^	100.00 ± 6.99 ^B^	130.00 ± 8.37 ^A^

Note: Letters indicate that there is a significant difference between data in the same row. PBP1 indicates whole-cut plant-based pork1, PBP2 indicates whole-cut plant-based pork2, PBP3 indicates whole-cut plant-based pork3, C-PBP1 and C-PBP2 indicate two commercially available plant-based pork products, and APM indicates animal pork meat.

## Data Availability

Not applicable.
